# Face coverings increase apparent honesty and cooperativeness

**DOI:** 10.1038/s41598-023-49127-9

**Published:** 2023-12-15

**Authors:** Janek S. Lobmaier, Daria Knoch

**Affiliations:** Department of Social Neuroscience and Social Psychology, Institute of Psychology, University of Bern, Bern, Switzerland

**Keywords:** Psychology, Human behaviour

## Abstract

People readily make inferences about trait-like characteristics of another person’s face. Since the recent global COVID-19 pandemic, the widespread use of hygienic face masks has led to large proportions of the face being covered. We investigated the effect of face masks on the inference of prosocially relevant characteristics, namely cooperativeness and honesty. Portraits of participants of previous studies from which we knew their “true” prosocial tendencies served as stimuli. These facial stimuli were presented once with and once without a hygienic face mask to 60 naïve participants who rated the faces for cooperativeness and honesty. Results revealed that wearing face masks made people generally appear more cooperative and more honest than without a mask, but that these ratings were unrelated to the true prosocial tendencies of these people. Together, these findings have important implications for social interactions, particularly in contexts where nonverbal communication is essential, such as in healthcare settings, job interviews, and social gatherings.

## Introduction

People automatically draw trait inferences from the facial appearance of others. Such first impressions are formed automatically and very fast: a 100-ms exposure time has been shown to be sufficient for participants to form an impression^1^. While these trait attributions are not necessarily accurate, it is noteworthy that humans typically show high agreement on trait inferences made based on facial appearance^2–4^. First impressions are inevitably influenced by stereotypes and biases^5^ and although collective wisdom warns against judging "a book by its cover", people seem unable to suppress this tendency. The fact that we all automatically form first impressions and that we typically agree on the characteristics we infer from facial appearances makes this a highly relevant field of research. Meanwhile, since the recent COVID-19 pandemic, the use of hygienic face masks has become much more commonplace in western parts of the world. Because face masks cover a large part of the face, they make the perception of social cues in faces more difficult, raising the question whether face masks influence the attribution of socially relevant characteristics.

First impressions can have a marked influence on subsequent interactions. For example, in almost every domain of life attractive people are treated more favourably than unattractive people^6–8^. Furthermore, baby-faced individuals are less likely to receive severe judicial sentences than more mature-faced individuals^9^. Initial facial impressions have also been shown to affect election outcomes^10^. These findings suggest that facial appearance can influence important personal decisions and can have significant social consequences.

Whether human beings can reliably identify socially relevant traits solely from visible cues in the static, non-expressive face is less unambiguous. Some researchers believe that certain personality characteristics such as conscientiousness or extraversion^11,12^, sociosexuality^13^, trustworthiness^14^, and aggression^15^ can be recognized somewhat accurately. Nonetheless, while research has shown consensus in perceptions of apparent traits, evidence for validity in these trait judgements remains patchy.

Inferences of trait-like characteristics depend on various facial features and are constructed from several visual cues. Facial features that people make use of when forming first impressions include the shape of the face, eyebrows, eyes, mouth, nose, cheekbones, and facial width-to-height ratio^14,16–19^.

After the recent outbreak of the global COVID 19 pandemic, the use of hygienic face masks has become more frequent. Because face masks cover a major part of the face, they substantially impair face perception^20–22^ and emotion recognition^21–25^, but less so fundamental mechanisms underlying social behaviours such as gaze cueing and gaze perception^26,27^. Consequently, the use of face masks also obscures the information from which we make inferences of trait-like characteristics. The difficulty to correctly “read” faces as a result of face covering leads to detrimental effects on various aspects of social cognition, such as establishing and maintaining effective interpersonal social interactions^28,29^. The impact of face masks on inferences of socially relevant characteristics is hence an important question, as it has implications for social interactions, particularly in contexts where nonverbal communication is essential, such as in healthcare settings, job interviews, and social gatherings.

To date, only few studies have examined the effect of face masks on the inference of socially relevant characteristics, with mixed results. Some studies suggest that face masks may have a positive effect on first impressions, leading to increased perceived attractiveness and likeability of the wearer^30,31^. Looking more specifically into how face masks impact the inferences of prosocial characteristics, Oldmeadow and Koch found that wearing face masks increases the perceived trustworthiness of others^32^. Similarly, Cartaud and colleagues^33^ found that people were perceived as more trustworthy when wearing face masks, which in turn lead to reduced preferred interpersonal distance. Lau^34^ found that masked faces not only appeared to be more trustworthy, but also younger, more attractive, and more approachable than unmasked faces. Occluding the bottom half of a face by means of a face mask seems to affect the ability to discriminate facial trustworthiness more strongly than the ability to discriminate facial dominance^35^. Contrary to these findings, Twele and colleagues report that face masks have a somewhat limited influence on first impressions of trustworthiness^36^ and Bylianto and Chan^37^ found that the presence of face masks actually leads to less approachability and trustworthiness ratings (see also^38^). Merging above mentioned findings suggests that face masks sometimes, but not always result in more favourable ratings, at least for trustworthiness. No study has yet looked at cooperativeness and honesty. Moreover, the studies mentioned above used faces from face databases (e.g.,^32,34,37^) or used computer generated faces^35^ which were rated for trait-like characteristics, without knowing the “true” characteristics of these people.

Even after having overcome the COVID-19 pandemic, the use of hygienic face masks is expected to continue to be part of normality in our everyday lives^39,40^. Hence, evidence-based research investigating the influence of mask wearing on social interactions is still needed. The present study aims to contribute to this knowledge by examining the impact of face masks on trait-like characteristics of prosociality (cooperation and honesty) in a controlled laboratory setting. Notably, and in contrast to previous studies, we used faces as stimuli of which we knew the real behavioural tendency to act in a more or less prosocial manner, rather than presenting images of random people. Participants were asked to evaluate a series of facial photographs on the prosocial dimensions “cooperativeness” and “honesty”. The photographs stemmed from participants of previous studies looking at individual differences in social behaviour. In these previous studies, the participants played the public goods game (PGG)^41^; to measure cooperativeness, or the temptation to lie card game (card deception game, CDG)^42^, which measured the propensity to behave in a (dis)honest manner. We hence had authentic behavioural measures of how cooperative (PGG) and how honest (CDG) these former participants were. Photographs of the faces of these former participants were presented with and without face masks and were rated for honesty and cooperativeness. Because attractiveness is known to influence judgements of socially relevant characteristics (e.g.,^7,8^), all stimuli were additionally rated for attractiveness. With this study we try to establish how face masks affect inferences of cooperativeness and honesty, and whether our inferences correspond to the true prosocial characteristics of a person.

## Methods

### Participants

A total of 60 participants (16 men, 44 women) aged between 18 and 32 years (mean age = 23.7; SD = 3.21) volunteered to take part in this study for course credit or a snack. There were no exclusion criteria. Fifty-two participants self-identified as being heterosexual, one person indicated to be homo- and seven to be bisexual.

The research was approved by the ethics committee of the Faculty of Human Sciences of the University of Bern (approval number: 2022-02-00006) and participants were treated according to The Code of Ethics of the World Medical Association (Declaration of Helsinki). All participants gave written informed consent and were informed of their right to discontinue participation at any time. Data were collected in a single wave between April and May 2022 and then analysed (no analyses were calculated before all participants were tested). At this time, mask wearing was no longer mandatory in Switzerland, but it was much more common-place than before the COVID-19 pandemic.

### Stimuli

The stimuli depicted faces of male participants who took part in previous studies^41,42^. Gianotti et al.^41^ used a public goods game (PGG) in which participants played in groups of four. Each participant was endowed with 20 monetary units (MU) and was asked to indicate how many MU they were willing to contribute to a public good. Each contributed MU was multiplied by 2 by the experimenter and was then split equally among the four group members. Participant’s earning consisted of all the MUs returned from the public good as well as the MUs not contributed in the first place. The size of the contribution served as our measure for cooperative behaviour.

Globig et al.^42^ adopted the temptation to lie card game (card deception game, CDG) to measure spontaneous (dis)honest behaviour. In this two-player/two-card game, the player receiving the ace of spades loses, whereas the player receiving the ace of hearts wins. Player A was the first mover and chose one of two covered cards without knowing the outcome of that choice. Player B was then presented with the uncovered cards. Unlike Player A, Player B thus knew the outcome of Player A’s choice. Player B then revealed the outcome of Player A’s choice to Player A. Importantly, Player B was explicitly informed that he could reverse Player A’s choice by lying to the latter about which card they had received. Player B could hence either accept the card he had been assigned or choose the other card, thereby lying to Player A about the outcome of the trial. The number of lies in this game was used as a behavioural measure of honesty (i.e., less lies, more honest).

**Figure 1:**
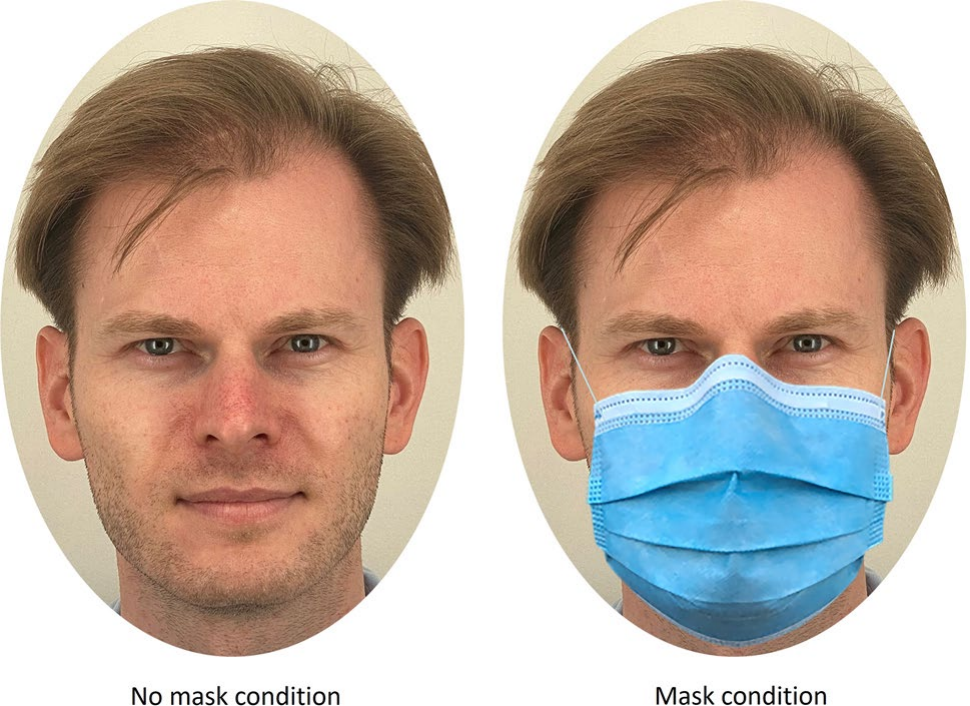
Example stimulus, left without a face mask, right with a face mask.

To exclude any effects of gender, ethnicity or age, the images were limited to faces of young Caucasian men, aged between 18 and 37 years (mean age = 22.7 years). For each of the two prosocial dimensions (cooperative and honest behaviour), the faces of the ten participants with the highest behavioural score on the respective characteristic and the ten with the lowest behavioural score were selected, resulting in 20 faces per characteristic. Hygiene masks were applied to the faces using Adobe Photoshop. Each face was shown to the participants once without and once with a face mask. A stimulus example is shown in Fig. 1.

### Task and procedure

After obtaining written informed consent, participants were seated comfortably in a dimly lit room and received written instructions for the tasks. They sat at a distance of approximately 60 cm from a PC screen. The face stimuli appeared on the screen with a width of 7 cm, thus subtending a visual angle of approximately 6.7°. This corresponds to a distance of approximately 140 cm in real life. Lighting conditions were kept constant for all participants. The study was implemented with Qualtrics. The actual experiment started by presenting brief on-screen instructions that participants will be seeing a series of faces and that they would be required to rate each face on the dimension of cooperative or honest behaviour, depending on the block. Half of the faces wore face masks, the other half did not. Each trial consisted of a face and below it, a visual-analogue slider bar with the underlying range between 0 and 100 with the endpoints labelled as “not at all cooperative”/“very cooperative” and “not at all honest”/“very honest”, respectively. The face was presented until the rating was given and the “next” button was pressed using the computer mouse. Participants were asked to submit their ratings as quickly as possible without pondering too long on their answers. Each block comprised 40 trials [20 facial identities (ten scoring low on the respective prosocial dimension and 10 scoring high) × 2 conditions (mask vs. no mask)]. Masked and unmasked stimuli were presented randomly within each block and the order of blocks was randomized between participants.

After the two rating tasks which each took approximately 5–10 min to complete, participants filled in a short demographic questionnaire assessing handedness, education, relationship status, sexual orientation, and gender of the participant. The experiment was completed by a final block in which participants were asked to rate all previously presented faces (with and without mask) for attractiveness. Again these faces were presented in a random order. After completing the attractiveness rating, participants were debriefed, thanked and dismissed.

### Statistical analyses

Linear mixed models (LMMs) were run using the R package lme4^43^, while the package lmerTest^44^ was used to determine the significance of predictors and random effects. All models were estimated using restricted maximum likelihood (REML). As recommended by Luke^45^, Satterthwaite's degree of freedom method was used to assess the significance of fixed effects. To balance type I and type II errors, a modelling approach was used to determine the appropriate structure of the random effects^46^. For each model, we started with the maximum random effect structure allowed by the design and progressively deleted the non-significant variance components. The significance of the random effects was determined by a likelihood ratio test. Convergence issues were addressed by simplifying the random effect structure. However, to calculate the explained variance (R2) by mask condition, we relied on a random intercept model, as suggested by LaHuis et al.^47^. Continuous predictors were z-transformed to improve convergence properties.

The analysis of the two prosociality indices (cooperativeness and honesty) was carried out using the same procedure. As each participant rated each face once, with and without a mask, the data were modelled with crossed random factors for participant ID and stimulus ID. To assess the need for an LMM, we first calculated the intraclass correlation (ICC) of each random factor with a random intercept model (Model 1 for cooperativeness and Model 6 for honesty). The calculated ICC corresponds to the ICC(1) by the notation of McGraw and Wong^48^. We then followed a simple to complex modelling approach. The base model included only the effect of the mask condition on prosociality judgments (Model 2 and Model 7). The mask variable was dummy coded with mask as the reference category. In a next model (Model 3 and Model 8), we included the 'true' prosociality trait of the stimulus person (FaceID), namely the amount the person contributed to the public good in the PGG (i.e. cooperativeness) or the number of times the person lied in the CDG (i.e. honesty). This allowed us to test whether the prosociality ratings corresponded to the actual prosociality scores. To check the robustness of the results, we included the covariate of participants' gender (Model 4 and Model 9) and face attractiveness ratings (Model 5 and Model 10) in two successive models. Thus, the final full Model 5 and 10 included the predictors attractiveness ratings, participant gender, true prosocial trait (cooperativeness or honesty), and mask condition. No violations of model assumptions were observed by visual inspection of residuals and random effects plots (R package).

To explore interrater agreement, we assessed the interrater reliability using the psych R package^49^. We calculated Cronbach’s alpha, the most widely used measure of interrater-reliability^50^. In addition, we calculated the ICC, based on a single measurement, absolute agreement, 2-way random effects model, as suggested by Williams and Apicella^51^. This ICC corresponds to the ICC(A,1) by the notation of McGraw and Wong^48^.

### Informed consent

Informed consent has been obtained from the participant modelling as example stimulus (Fig. 1) for publication of identifying images.

## Results

### Cooperativeness (PGG)

#### Linear mixed models

The intercept-only model (Model 1) revealed an ICC of 0.088 for PartID and an ICC of 0.094 for FaceID, meaning that 8.9% of the total variance in the cooperation ratings was due to differences between individuals and differences in stimuli account for 9.4% of the total variance. The inter-individual differences between participants (PartID: LR = 141.76; *p* < 0.001), as well as the variance between FaceIDs (FaceID: LR = 194.70; *p* < 0.001), were significant, indicating the need to account for these random factors. Model 2 revealed a significant mask effect (Estimate: − 9.87; CI − 11.43 to − 8.31; p < 0.001), whereby 6.3% of the level-1 variance of prosociality rating was explained by mask condition. Thus, faces without a mask were rated -9.87 points less prosocial. Random slopes were estimated for the mask condition on both random factors (PartID and FaceID).

When adding “true” cooperative behaviour to the model (Model 3), the effect of mask remained significant (Estimate: − 9.83; CI − 14.03 to − 5.63; p < 0.001), whereas true cooperativeness did not predict cooperativeness ratings (Estimate; 0.81; CI − 2.37 to 3.98; p = 0.618). Adding participant sex to the model (Model 4) did not markedly change the mask effect (Estimate: − 9.82; CI − 14.03 to − 5.62; p < 0.001), while neither true cooperative behaviour (Estimate: 1.31; CI − 1.63 to 4.25; p = 0.382) nor participant sex (Estimate: − 1.71; CI − 6.10 to 2.67; p = 0.444) were statically significant predictors.

**Figure 2:**
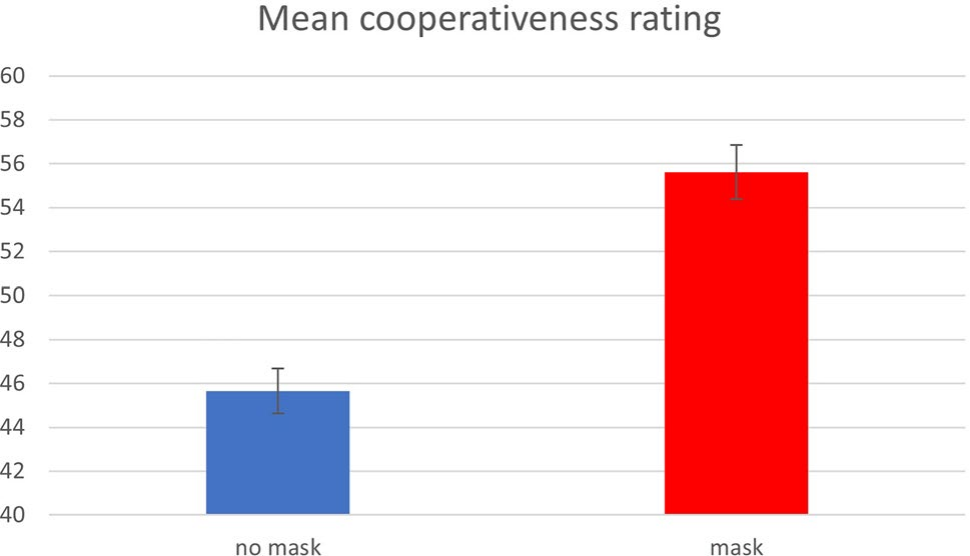
Mean cooperativeness ratings in the no mask condition (left) and the mask condition (right). Error bars depict standard errors of the mean (SEM).

The full model (Modell 5) revealed that attractiveness significantly predicted rated cooperative behaviour, (Estimate: 6.74, CI 5.53–7.96; p < 0.001). While the effect of the mask condition was reduced but still significant, participants' sex and true cooperative behaviour remained insignificant (see Table 1). The main effect of mask condition is visualised in Fig. 2.

**Table 1:** LMM results for the cooperativeness ratings (Model 5).

Predictors	Full model “cooperativeness rating”		
	Estimates	CI	P
(Intercept)	41.74	36.24–47.23	** < 0.001**
condition [nomask]	− 8.22	− 12.06 to − 4.39	** < 0.001**
partsex [m]	− 1.54	− 5.27 to 2.19	0.418
PGG (z)	0.57	− 2.16 to 3.31	0.680
Attractiveness (z)	6.74	5.53–7.96	** < 0.001**

Observations: 2329; Marginal R^2^/Conditional R^2^: 0.143/0.351.

PGG (z): z-transformed observed behavioural value in the public goods game (“true” behaviour); Estimates: regression coefficients; CI: confidence interval; *P*: p-value.

Significant values are in bold.

#### Interrater reliability

For masked faces, Cronbach's α ratings for cooperativeness was α = 0.919 and for faces without hygiene masks α = 0.909, corresponding to an excellent agreement. The ICC calculations suggested a low inter-rater agreement for both the mask condition (ICC = 0.13, 95% CI [0.08, 0.26]) and the no mask condition (ICC = 0.13, 95% CI [0.07, 0.25]).

### Honesty (CDG)

#### Linear mixed models

The intercept-only model (Model 6) revealed an ICC of 0.068 for PartID and an ICC of 0.119 for FaceID, meaning that 6.8% of the total variance in the honesty ratings was due to differences between individuals and differences in stimuli account for 11.9% of the total variance. Again, both random intercepts were significant (PartID: LR = 101.58; *p* < 0.001; FaceID: LR = 252.49; *p* < 0.001).

Model 7 revealed a significant mask effect (Estimate: − 4.29; CI − 5.92 to − 2.67; p < 0.001), whereby 1.1% of the level-1 variance of honesty rating was explained by mask condition.

When adding “true” honesty to the model (Model 8), the effect of mask remained significant (Estimate: − 4.25; CI − 8.25 to − 0.26; p = 0.037), but true honesty did not predict the honesty ratings (Estimate; − 0.26; CI − 3.59 to 3.07; p = 0.878). Adding participant sex to the model (Model 9) hardly changed the mask effect (Estimate: − 4.25; CI − 8.25 to − 0.26; p < 0.037), while neither “true” honesty (Estimate: − 0.26; CI − 3.59 to 3.07; p = 0.879) nor participant sex (Estimate: 0.73; CI − 3.11 to 4.58; p = 0.708) were statically significant predictors.

**Figure 3:**
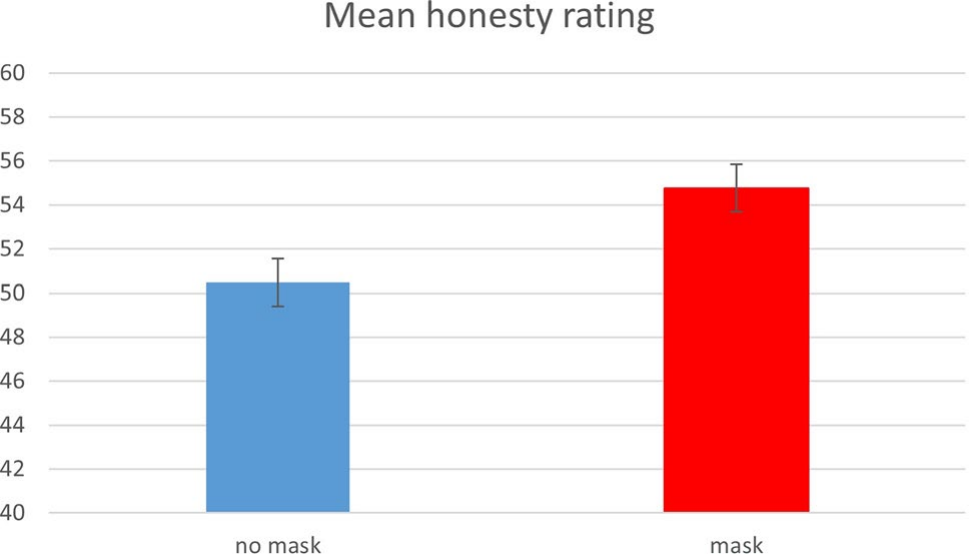
Mean honesty ratings in the no mask condition (left) and the mask condition (right). Error bars depict standard errors of the mean (SEM).

The full model (Model 10) revealed that attractiveness significantly predicted rated honesty, (Estimate: 5.14, CI 3.66–6.63; p < 0.001). Mask condition just failed to remain a significant predictor and participant sex and true honesty were again insignificant predictors (see Table 2). The main effect of mask condition is visualised in Fig. 3.

**Table 2:** LMM results for the honesty ratings (Model 10).

Predictors	Full model “honesty rating”		
	Estimates	CI	P
(Intercept)	45.07	39.43–50.71	** < 0.001**
condition [nomask]	− 3.43	− 6.91 to 0.05	0.054
partsex [m]	0.47	− 3.09 to 4.03	0.795
CDG (z)	0.15	− 2.81 to 3.11	0.919
Attractiveness (z)	5.14	3.66–6.63	** < 0.001**

Observations: 2335; Marginal R^2^/Conditional R^2^: 0.061/0.279.

CDG (z): z-transformed observed behavioural value in the card deception game (“true” behaviour); Estimates: regression coefficients; CI: confidence interval; *P*: p-value.

Significant values are in bold.

#### Interrater reliability

For masked faces, Cronbach's α ratings for cooperativeness was α = 0.896 and for faces without hygiene masks α = 0.934 corresponding to an excellent agreement. Again, the ICC calculations suggested a low inter-rater agreement for both the mask condition (ICC = 0.11; 95% CI [0.06, 0.22]) and the no mask condition (ICC = 0.17; 95% CI [0.01, 0.32]).

## Discussion

Previous research suggests that people readily draw inferences of socially relevant traits based on visible cues in the static, nonexpressive face. Meanwhile, the use of hygienic face masks has drastically increased since the COVID-19 pandemic, raising the question whether and how face-covering influences trait inferences based on first impressions. In the present study we investigated the effect of face masks on inferences of prosocial characteristics made from faces. Notably, we also tested whether the true prosocial tendency of a person reliably predicts prosocial trait attributions based solely on the facial appearance. To do so we presented observers with portraits of men of whom we knew their actual cooperative and honest behaviour from previous studies. Each face was once presented wearing a hygienic face mask and once without wearing a mask. We found that wearing hygienic face masks made people generally appear more cooperative and more honest than without a mask, but that these ratings were unrelated to the true prosocial tendencies of these people.

Our findings suggest that when people cover a large part of their face with a hygienic face mask, they appear to have more favourable characteristics (i.e., more honest, more cooperative) compared to when their whole face is visible. This finding parallels previous reports, suggesting that wearing a face mask has a positive effect on perceived trustworthiness of others^32,33^ and that masked faces appear younger, more attractive, and more approachable than unmasked faces^34^. Further analyses of the present results revealed that the mask-induced increase in favourable characteristics can be partly ascribed to the fact that the faces appeared to be more attractive when wearing a mask. This again is in line with a growing number of recent studies reporting perceptions of increased attractiveness in masked faces^27,30,31^. Overall, the incidental impact of face masks on facial attractiveness can contribute to a more favourable perception of an individual's face, which in turn serves as a halo^52^ for other favourable characteristics, such as honesty and cooperativeness.

While our results clearly demonstrate that individuals wearing masks are perceived as more honest and cooperative, we can only speculate about the underlying mechanisms at play. Possibly, people wearing face masks appear more favourable, because wearing a mask can be seen a prosocial act (e.g., protecting other people in the vicinity from contagion). Alternatively, wearing a face mask might generally increase ambiguity, which then leads to an uncertainty bias when encountering a person wearing a mask. As a result, this bias may prompt people to give higher ratings to faces with masks. Whether people wearing face masks generally appear more favourable or whether the higher ratings are a result of an uncertainty bias will have to be ascertained in future studies.

Our findings suggest that the apparent prosocial characteristic inferred from a face does not correspond to the “true” characteristic of the person. The idea that humans can reliably and accurately identify socially relevant traits solely from visible cues in the face has occupied mankind since time immemorial. While such an ability seems beguiling and arguably would lead to evolutionary and adaptive benefits, we find no evidence for the skill to accurately discern people’s prosocial characteristics from their faces. Yet, regardless of the defective propensity to infer trait-like characteristics from faces, we all seem to do this with great ease and without evil intent. It seems that we overestimate our abilities and, paradoxically, draw the wrong conclusions. In order to assess our counterparts as quickly as possible, we try to interpret visual cues and generalise subjective experiences. The information gained can help to assess the immediate behaviour and intentions of the counterpart, but, as we demonstrate here, it does not serve as a direct source of information about a person's prosocial traits (cf.^53^).

We note that the present study was conducted during a time after the peak of the global COVID-19 pandemic, when face masks were still common-place but no longer mandatory. Undoubtedly, the social meaning of wearing a face mask had changed with the pandemic. Our results should therefore be interpreted in the context of the COVID-19 pandemic, during which mask-wearing was strongly recommended as a safety-measure to reduce the spread of the disease. We cannot be sure that the same results would have occurred, had we conducted the study in a time that is more distant to the COVID-19 outbreak.

Taken together, we found that wearing a face mask significantly increases apparent cooperativeness and honesty of a person. This might be explained by the fact that masked faces appear to be more attractive than unmasked faces, and that this increase in apparent attractiveness serves as a halo for other trait attributions. Importantly however, we found no evidence that these trait inferences are actually reliable, neither when the target person is wearing a mask nor when their face is fully visible. These findings have important implications for social interactions, particularly in contexts where nonverbal communication is essential, such as in healthcare settings, job interviews, and social gatherings. By realising that face masks positively affect inferences of cooperativeness and honesty, we may be better equipped to navigate social interactions in the context of future pandemics.

### Supplementary Information

Supplementary Information.

## Data Availability

The data of this study are included as Supplementary Information file.
